# Intestinal Obstruction in a Child with Massive Ascariasis

**DOI:** 10.1155/2021/8857291

**Published:** 2021-01-08

**Authors:** Munanura Turyasiima, Paul Matovu, Gloria Kiconco, Walufu Ivan Egesa, Phillip Sunday, Lydia Nakandi, Kirya Musa, Denis Oluka, Martin Byendera

**Affiliations:** ^1^Pediatrics and Child Health Department, Kisiizi Hospital Church of Uganda, Kabale, Uganda; ^2^Pediatrics and Child Health Department, Kampala International University, Western Campus, Bushenyi, Uganda; ^3^Surgery Department, Kisiizi Hospital Church of Uganda, Kabale, Uganda

## Abstract

Soil-transmitted helminths are so prevalent in the tropics and low developing countries. Pediatric clinical presentation of ascariasis, the most common helminth, as the intestinal obstruction is not only rare but also less described. We present a case of a 4-year-old girl with massive ascariasis. She presented with a 3-day history of acute abdominal pain associated with vomiting and an episode of passing long white roundworms, about 5 cm in length, through the nose. The child had mild constipation and passed pellets of hard stool once in the last 72 hours. She was in fair general condition at the examination but had significant findings on abdominal examination. On palpation, there was a soft mass localized in the left paraumbilical area and no tenderness, with normal bowel sounds on auscultation. Exploratory laparotomy was sanctioned where roundworms (*Ascaris lumbricoides*), saucepan full, were delivered through a 2.5 cm enterotomy incision. Postoperative management was carried out, and the child discharged on the 7^th^ day of treatment including a 3-day course of albendazole 400 mg daily.

## 1. Introduction

Soil-transmitted helminths (STH) infect about 1.5 billion people worldwide [1] and cause short- and long-term effects, especially among children. *Ascaris lumbricoides* is among the most prevalent of all other different species of the STH in most parts of the world including Uganda [2–5]. We present a child who presented with an acute intestinal obstruction that was at admission misdiagnosed as intestinal intussusception, but later discovered to have massive ascariasis during an exploratory laparotomy.

## 2. Case Presentation

### 2.1. Sociodemographic Information

A. L is a 4-year-old girl from Nyakishenyi subcounty, Rukungiri district, a rural remote setting in southwestern Uganda, who was admitted to the pediatric ward of Kisiizi Hospital, in July 2020.

### 2.2. History

She presented with a 3-day history of abdominal pain and vomiting episodes. The abdominal pain was of gradual onset, severe, and localized in the umbilical area, radiating to the lower abdomen. She had three vomiting episodes that were nonbilious, containing food contents, and could occasionally pass out long white roundworms, about 5 cm in length through the nose. She had mild constipation, passing pellets of hard stool. She also reported on and off low-grade fever. The other system reviews were unremarkable. She had no chronic medical disease and was HIV/AIDs negative. The child had been only dewormed once in the last 18 months. Growth and development milestones were normal according to the mother.

### 2.3. Physical Examination

We examined a preschool-going girl in a fair general condition, afebrile (axillary temperature of 36.7°C), not in distress, with no pallor, no lymphadenopathy, no dehydration, no edema, and no clubbing. The child had a good nutrition status, and all systemic examinations were normal except the abdominal exam that had significant findings. There was no distension on inspection, and she had a palpable mass of about 4 cm in length localized in the left paraumbilical area, soft, mobile, and nontender, no organomegaly, and had normal bowel sounds on auscultation. The initial differential diagnoses included intussusception and mesenteric cyst.

### 2.4. Laboratory and Imaging

Complete blood counts, urinalysis, stool microscopy, and serum electrolytes were conducted and turned out normal. An ultrasound scan revealed a round mass in the right iliac region that made the team think of intussusception, with all other internal organs normal. Chest radiography was performed and turned out normal.

### 2.5. Surgical Interventions

**Figure 1:**
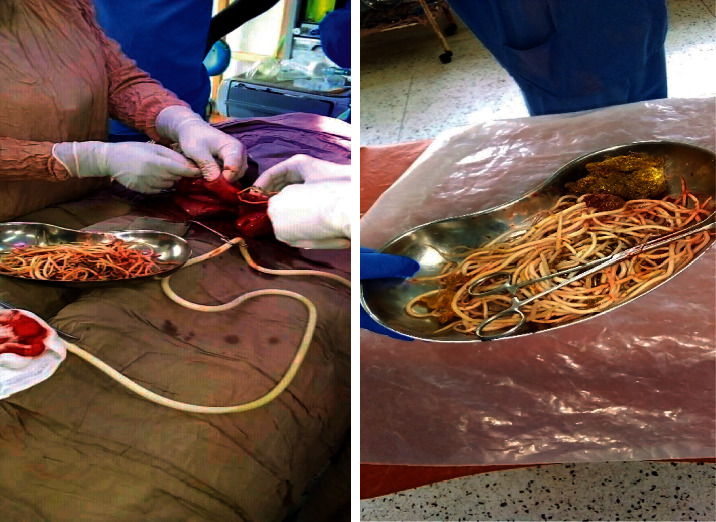
Physical appearance of *Ascaris Lumbricoides* extracts during exploratory laparotomy.

The child was admitted and rehydrated with 2L of Ringers Lactate and 1L of 5% dextrose on the first day, and prophylactic antibiotics (IV ceftriaxone 50 mg/kg) were given 1 hr before surgery. On the 2^nd^ day of admission, an exploratory laparotomy was sanctioned. Using the surgical approach through a subumbilical transverse abdominal incision, a 2.5 cm enterotomy was made in the distal part of the ileum just before the ileocecal junction and collected a saucepan full of roundworms by manually extracting them and milking the bowel. The roundworms were live, white in color, averagely 10–15 cm in length, the longest measuring 25 cm long, causing complete obstruction of the ileum.  Figure 1 shows the physical appearance of *Ascaris lumbricoides* extracts during exploratory laparotomy.

### 2.6. Medical Treatment

Postoperation management with intravenous fluids (IV normal saline for 24 hours), prophylactic antibiotics (IV ceftriaxone 50 mg/kg/day, and IV metronidazole 15 mg/kg/8hrly for 3 days), and antihelminthic drugs (oral albendazole 400 mg daily for 3 days) was administered.

### 2.7. Outcomes

Feeding was reinitiated gradually starting with oral sips at 24 hours after operation until normal feeding on the 7^th^ day after operation when discharge was planned. At discharge, she was in fair general condition, feeding, passing stool and normally ambulating. Abdominal stitches were removed, and the child was allowed home with a plan for review in a 2 weeks' period.

### 2.8. Discharge Plan

Health education on the risk factors and prevention of STH infestations was given to the parents by the medical team. At discharge, the mother was dewormed with a single dose of albendazole (400 mg). The child was reviewed after 2 weeks at the pediatric clinic, where a control stool microscopy turned normal with no ova/cysts of *A. lumbricoides* found. Her sibling, a 28-month-old male, was also brought for routine deworming, and all encouraged to continue the mandatory semiannual deworming every 6 months thereafter.

## 3. Discussion

Massive ascariasis is a term used for repeated autoinfection by roundworms that then allows the worms to multiply to overwhelming numbers. This is common in persons with weakened immune system and/or other at-risk groups, especially among preschool- and school-going children. *Ascaris lumbricoides* massive infestation is associated with many complications including undernutrition due to anorexia, psychosocial disability, and mortality. The larvae can spread to other organs such as the lungs, liver, and/or spleen. The worms in massive numbers obstruct the intestinal lumen to cause intestinal obstruction, the process that happened in this case. A mortality case autopsy report in the 20^th^ century described massive ascariasis with intestinal and extraintestinal fatal manifestations in a 2-year-old South African child who presented with vague abdominal symptoms [6].

Soil-transmitted helminth (STH) infections are prevalent in low-resource setting communities where hygiene and sanitation are poor [1, 2]. The key factors linked with a higher prevalence include poor socioeconomic conditions, poor hand hygiene, sanitation practices such as not washing hands after defecation [3, 7–9], and maternal STH infections during pregnancy [4]. Even though infection can occur at any age, the highest rate is in preschool- or early school-age children [10]. This child is a preschool girl with poor socioeconomic background, but also missed the routine deworming program that could have increased the risk of massive ascariasis.

**Figure 2:**
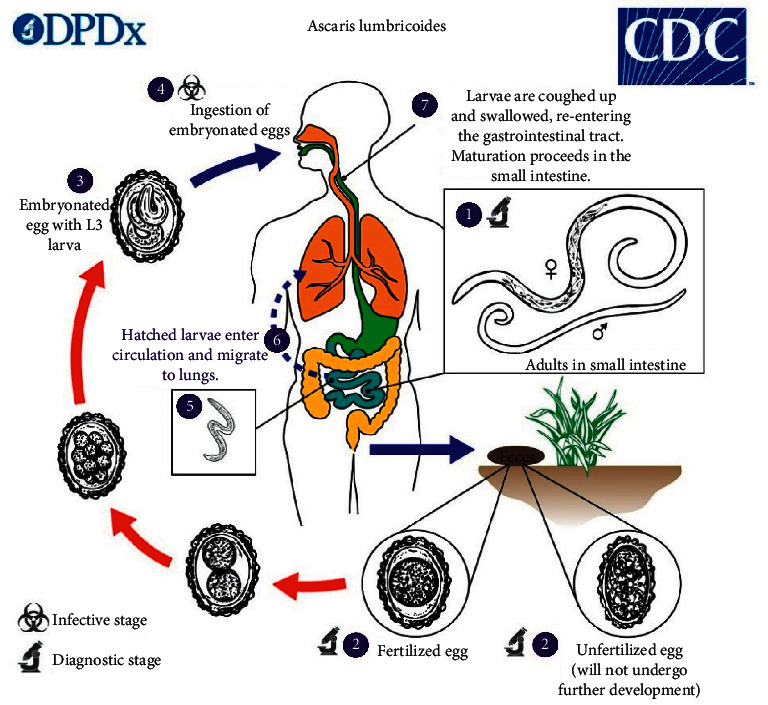
The life cycle of *Ascaris lumbricoides* summarized (source: Centers for Disease Control and Prevention).

Humans and swine are the main hosts for *Ascaris lumbricoides*. Adult worms living in these hosts lay eggs that later hatch into larvae, which through systemic circulation reach the lungs where they get access to the stomach; this is summarized in Figure 2. Transmission can be through the oral-fecal route and/or skin penetration. This process causes autoinfection and makes the infection to become massive if no treatment is given. The life cycle of *A. lumbricoides* explains how fast these worms can multiply and cause massive ascariasis, especially in immunocompromised children. This child had neither immunosuppressive disease nor immunosuppressive treatment but had not been dewormed for the last 8 months.

A high index of suspicion is needed to diagnose pulmonary ascariasis or obstruction of the gastrointestinal tract in the clinical context. Two diagnostic methods including conventional and molecular methods are currently available. Conventional methods include microscopy, culture, and egg counting. The rapid, highly sensitive molecular techniques, particularly quantitative polymerase chain reaction, make it suitable for diagnosing STH over insensitive, as well as labor-intensive conventional methods [11]. Unfortunately, the conventional methods are the most commonly used in low-resource settings. Ultrasound examination of the abdomen is capable of visualizing intraluminal adult worms. Stool microscopy and abdominal ultrasound scanning were the only immediate tests performed on this child and were normal. Definitive diagnosis was based on clinical evidence of roundworms in the vomitous, signs of intestinal obstruction, and surgical removal of the worms at exploratory laparotomy.

The WHO strategy for control of soil-transmitted helminth infections is to control morbidity through the periodic treatment of at-risk people living in endemic areas [1]. Ascariasis management involves primary prevention through proper hygiene and sanitation practices, periodic deworming, and specific treatment with antiparasitic medications when ova/cysts are discovered at microscopic stool analysis.

Uganda, through the ministry of health (MOH), runs a semiannual routine deworming program for all persons above 1 year of age, which has proven effective in significantly reducing helminthic infections in most of its districts [8, 9]. This program has been integrated into child health days or supplementation programs and/or school health days to increase access. This child had only received a single dose instead of three in the last 18 months before the presentation. The mother blamed the poor access to the public health facilities for the failure to take her children for the scheduled deworming. Control through periodical deworming to eliminate infecting worms, health education to prevent reinfection, and improved sanitation to reduce soil contamination with infective eggs can also reduce mortality and morbidity related to these infections [1]. The child's mother's knowledge of the risk factors and prevention of these infections was poor, and so preventive information was given to the caretakers as a discharge plan package.

## 4. Conclusions

Massive ascariasis should be considered as a differential diagnosis in children presenting with intestinal obstruction in clinical settings where soil-transmitted helminth infestations are prevalent. Public awareness and routine deworming, especially in the poor remote communities, through community health workers (village health teams), should be further strengthened to avoid complications associated with massive ascariasis.
